# Durability of Fibre Reinforced Polymers in Exposure to Dual Environment of Seawater Sea Sand Concrete and Seawater

**DOI:** 10.3390/ma15144967

**Published:** 2022-07-17

**Authors:** Fan Guo, Saad Al-Saadi, R. K. Singh Raman, Xiaoling Zhao

**Affiliations:** 1 Department of Mechanical and Aerospace Engineering, Monash University, Clayton, VIC 3800, Australia; faye.guo16@gmail.com (F.G.); saad.al-saadi@monash.edu (S.A.-S.); 2 Department of Chemical and Biological Engineering, Monash University, Clayton, VIC 3800, Australia; 3 Department of Civil Engineering, Monash University, Clayton, VIC 3800, Australia; zhao.xiao.ling@monash.edu; 4 Department of Civil and Environmental Engineering, The Hong Kong Polytechnic University, Hong Kong, China

**Keywords:** seawater sea sand concrete (SWSSC), carbon-fiber-reinforced polymer (CFRP), glass-fiber-reinforced polymer (GFRP), basalt-fiber-reinforced polymer (BFRP), moisture uptake, FTIR, SEM

## Abstract

The consequence of exposure to the dual environment of seawater sea sand concrete (SWSSC) on the inner surface and seawater (SW) on the outer surface on the durability of fibre reinforced plastic (FRP) confining tubes has received very limited research attention. The durability of FRPs fabricated with different fibre types was investigated for the application of SWSSC filled tubes and SWSSC-filled double-skin tubes exposed to the external environment of SW. The colour and shininess of carbon-fibre-reinforced polymer (CFRP) surfaces generally stayed unchanged even after 6 months of exposure to the dual environment, whereas basalt-fibre-reinforced polymer (BFRP) and glass-fibre-reinforced polymer (GFRP) tubes suffered degradation. The degradation led to a ~20–30% increase in pH; however, the pH increase in the external SW was more pronounced when the internal solution was SWSSC. The extent of degradation was greater in BFRP that in GFRP. The investigation also included a specialised investigation of the degradation at the fibre–matrix interface by fracturing specimens in liquid nitrogen.

## 1. Introduction

The global population is expected to increase by 2.25 billion over the next 40 years, and the world total could reach 9.15 billion in 2050 [1]. The immense population growth will necessitate a massive increase in infrastructure and construction materials such as concrete. The huge increase in demand of concrete will cause a shortage of its primary ingredients: water and river sand. There is a concern that the climate change may also trigger rising sea levels which will necessitate additional coastal infrastructures [2,3]; this will require the transport of large quantities of fresh water and river sand from inland to the costal sites. Excessive use of fresh water for concrete production adds to the concerns of water scarcity, whereas excessive dredging of river sand for this purpose is a concern for the river ecosystem. In this context, it is extremely attractive if the concrete could be produced using the locally available seawater and sea sand, instead of the fresh water and river sand. Therefore, there is great value in the investigation of aspects of seawater sea sand concrete (SWSSC). 

In spite of the enormous environmental, economic and social benefits of SWSSC, its reinforcement poses a great challenge since carbon steels that are traditionally used for the reinforcement of normal concrete will suffer unacceptably high corrosion rates due to the high Cl^−^ contents of seawater in SWSSC. Even in the case of normal concrete, the Cl^−^ ions that diffuse through to steel reinforcements are known be a serious concern since they slowly accumulate to deleterious amounts at the steel surface and disrupt the protective film, eventually causing spallation and cracking of concrete (that is also known as ‘Concrete Cancer’) [2,3,4]. 

It is essential to find a suitable alternative to steel reinforcement to mitigate the critical problem of unacceptably high corrosion rates of steel in SWSSC. FRPs are the composite materials that are potentially alternative materials for SWSSC reinforcements due to a set of advantages: high strength-to-weight ratio, high stiffness-to-weight ratio, good corrosion resistance and ease of installation. Some current practices of using FRPs as structural components in civil engineering include concrete reinforcing rods and concrete confining tubes, retrofitting wraps, composite bridge decks and all composite FRP structures [5].

**Figure 1:**
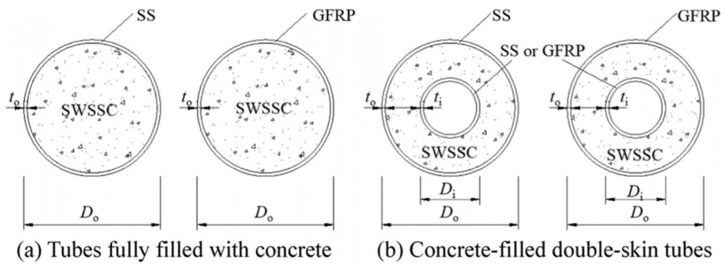
(**a**) concrete-filled tubes (CFT) and (**b**) concrete-filled double-skin tubes (CFDT). Used with permission from Thin-Walled Structures, Elsevier, 2016 [6].

**Figure 2:**
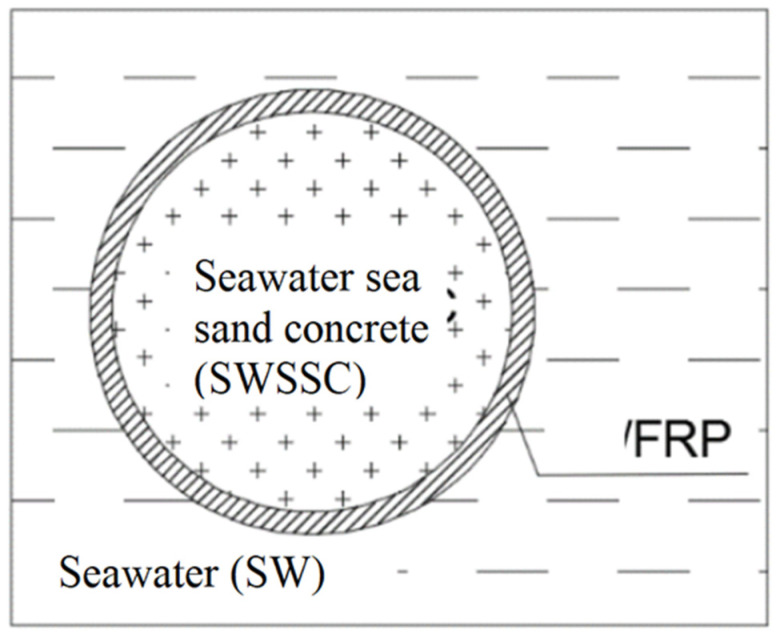
Exposure environment of SWSSC filled tubes.

Earlier studies on FRPs in aggressive environments focused on the durability of FRP reinforcing bars or laminates, whereas there is a paucity of studies on the durability of FRPs used in concrete-filled tubes (CFT) and concrete-filled double-skin tubes (CFDT). A CFT is a hybrid system that consists of an encasing tube and a core concrete (Figure 1a [6]). It can be used as piles or bridge piers in marine structures due to a range of advantages, that include excellent load-carrying capacity, lateral concrete confinement, permanent formwork and good seismic performance [7,8,9,10]. A CFDT is a special type of CFT that consists of an inner tube and an outer tube with concrete sandwiched in between (Figure 1b [6]), which can reduce the self-weight of the member and achieve greater stiffness [8]. Seawater sea sand concrete (SWSSC)-filled FRP tubes achieve greater strength than plain concrete and hollow section tubes [6,11]. However, there is little reported on the durability of FRP tubes used in such applications in the marine environment. This could be a concern because of the dual exposure environment, i.e., SWSSC internally and seawater (SW) externally, which could be more damaging than a single exposure environment of concrete pore solution (Figure 2).

There are a few reported studies [12,13,14] on the durability of filament-wound glass-fibre-reinforced polymer (GFRP) tubes that were immersed in a single exposure environment of tap water or seawater. A study [7] on GFRP tubes filled with conventional concrete immersed in artificial seawater at 50 °C for up to 1 year reported GFRP to demonstrate good durability performance as their tensile hoop strength was found to be retained at ~80%, and no significant microscopic degradations were observed in the cross-sections of polished and ground specimens.

**Figure 3:**
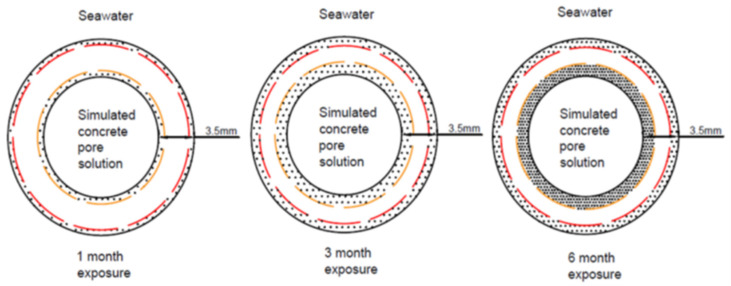
Hypothesised degradation pattern of a FRP tube exposed to simulated concrete pore solution internally and artificial seawater externally (Note: the intensity of dots represents the extent of degradation; yellow/red dash lines represent degradation fronts).

There is a need for a comprehensive investigation into the effect of a dual exposure environment of SWSSC internally, and SW externally (as seen in Figure 2) on the degradation of different types of confining FRP tubes, for the application of SWSSC-filled tubular/SWSSC-filled double-skin tubular structures in a marine environment. Exposure to such dual environment might lead to different degradation patterns, such as the one schematically shown in Figure 3, or a different degradation rate than those proposed for a single alkaline environment [15] or a single SWSSC environment [16] for internal FRP reinforcements. In the dual exposure environment (Figure 2), the internal surface of the tube is exposed to SWSSC, comprising a mix of alkali and chloride salts and the external exposure environment of seawater. Therefore, FRP constituents (i.e., fibre, resin, fibre–resin interface) adjacent to the internal surface might degrade to a severer extent (represented by the intensity of dots in Figure 3) and at a faster rate (represented by red/yellow dash lines for degradation fronts in Figure 3) in moving towards the mid-tube thickness than those adjacent to the external surface.

Therefore, based on the findings of the experimental investigation of FRPs in a single exposure environment of SWSSC [3], this study aims to systematically investigate the effect of the dual exposure environment of different simulated solutions of concrete and seawater sea sand concrete (SWSSC) internally (viz; normal concrete (NC), high performance concrete (HPC), seawater sea sand normal concrete (SWSSNC),and seawater sea sand high performance concrete (SWSSHPC)), and seawater externally, on the degradation of filament-wound tubes of a glass-fibre-reinforced polymer (GFRP), a basalt-fibre-reinforced polymer (BFRP) and a carbon-fibre-reinforced polymer (CFRP). The exposure to the simulated solution of seawater internally and externally is investigated as well.

## 2. Materials and Methods

### 2.1. Materials and Specimens

Filament-wound tubes of CFRP, BFRP and GFRP with a nominal outer diameter of 50 mm and a wall thickness of 3.5 mm were purchased from CST Composites, Ingleburn, Australia. The winding pattern is 20% 15° + 40% 45° + 40% 75°, and the epoxy resin in each FRP was a bisphenol A and bisphenol B blend with an amine hardener. Table 1 shows the chemical compositions of E-glass and basalt fibres that are present in FRPs used in the present work. The tubes were cut into short sections with a height of 35 mm. The dimension of each tubular specimen is 50 mm × 3.5 mm × 35 mm (i.e., outer diameter × thickness × height).

In order to create a dual exposure environment for tubular specimens, one side of the tube was firstly sealed onto a petri dish with an epoxy resin as the base, and the other side of the tube was sealed as well after the solution was filled inside (to ensure leak tightness). Tubular specimens were then placed in a container, which contains another type of solution, as required. All containers were held in a temperature-controlled water bath at 60 °C for up to 6 months.

### 2.2. Dual Exposure Environment

Four exposure combinations (Table 2) were used to simulate the real exposure environment of FRPs in concrete-filled tubes (CFT)/concrete-filled double-skin tubes (CFDT) (Figure 3). The combination C1 (i.e., seawater both as internal and external solution) was tested as a reference. Four simulated concrete solutions, i.e., normal concrete (NC), high performance concrete (HPC), seawater sea sand normal concrete (SWSSNC), and seawater sea sand high performance concrete (SWSSHPC), were used to simulate the internal concrete exposure environment of tubular specimens in the present experiment, and the chemical compositions of those solutions are presented in Table 3. The recipes of the simulated solutions used in the present study are described in literature [3,16,17,18,19,20]. Distilled water mixed with 35 g/L NaCl that simulates seawater (SW) was used as the external solution in all exposure combinations to simulate the external seawater exposure environment of CFT/CFDT structures.

After 3-month and 6-month exposures, the pH of both internal and external solutions was measured using a digital pH meter (Oakton pH700) for each of the different exposure combinations.

### 2.3. Scanning Electron Microscopy (SEM)

Scanning electron microscopy (SEM) observation and image analysis were performed on selected specimens after 6-month exposure to a given dual exposure environment using JEOL JSM-7001F FEGSEM (JEOL Ltd., Tokyo, Japan) to characterise the morphological changes at the edges and the centre of the specimens where the degradation fronts from two exposure surfaces could potentially meet. Specimens for SEM were prepared both by the grinding-and-polishing method and by fracturing the specimens after pre-exposure to liquid nitrogen, to examine the deterioration of the fibre and fibre–resin interface of composites after exposure. 

To address the mechanical damage that may happen to the interface during the grinding, the specimens were exposed to liquid nitrogen that embrittled the FRPs, thus enabling a brittle fracture along the fibre–matrix interface under an applied load and allowing SEM analysis of the interface. To enable SEM imaging, FRP specimens were coated with a thin layer of iridium for the required conductivity.

### 2.4. Attenuated Total Reflectance–Fourier Transform Infrared Spectroscopy (ATR-FTIR)

BRUKER-FTIR spectrometer (Bruker Corporation, Billerica, MA, USA) equipped with an attenuated total reflectance was used to study the chemical structures of unexposed FRPs. Three specimens were tested for each type of FRP for the examination of reproducibility.

After 6-month exposure at 60 °C, the residues of test solution from both the internal and external side of the tubular specimens in each case were collected for FTIR analysis to determine the leaching products from both dual exposure environments. FTIR measurements were carried out in the range of wave numbers from 4000 to 500 cm^−1^, and 64 scans were acquired with a spectral resolution of 4 cm^−1^. The details of the residue preparation and FTIR analysis procedure can be found in [3].

## 3. Results and Discussion

### 3.1. Visual Observation

**Figure 4:**
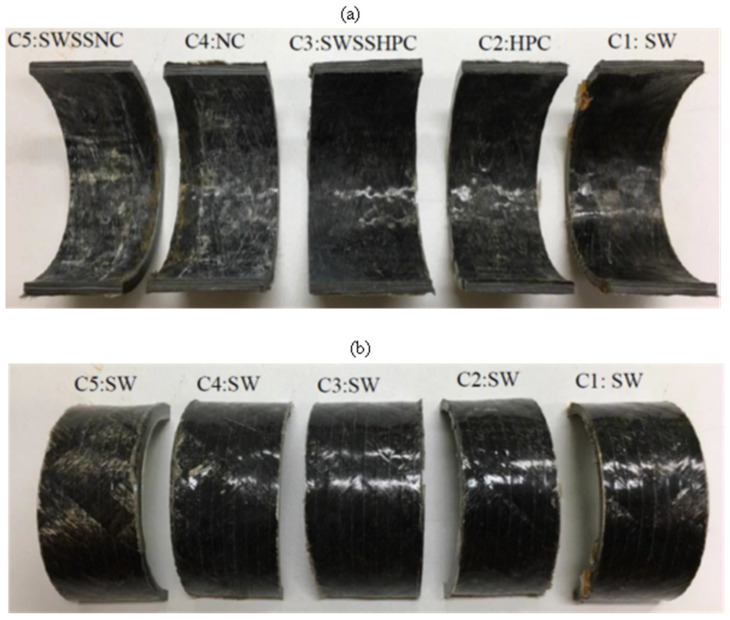
(**a**) Internal and (**b**) external surface change of BFRP tubes after 6-month exposure to the dual environments (Table 2).

**Figure 5:**
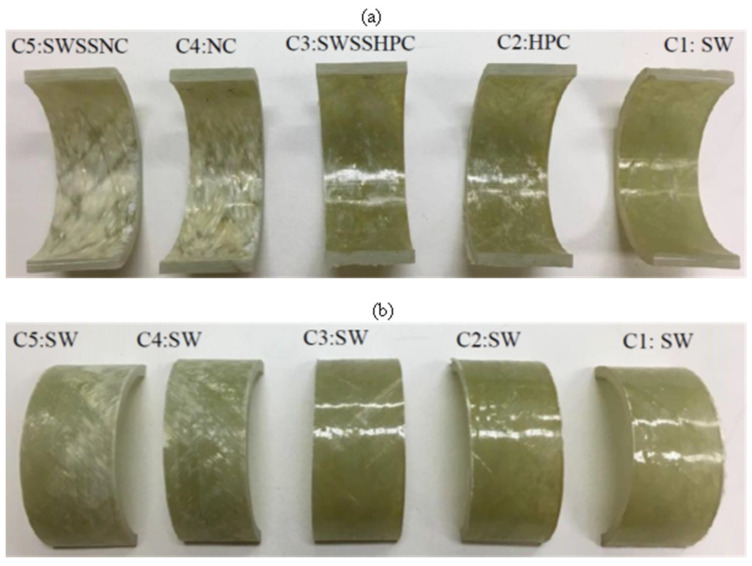
(**a**) Internal and (**b**) external surface change of GFRP (E-glass) tubes after 6-month exposure to the dual environments (Table 2).

**Figure 6:**
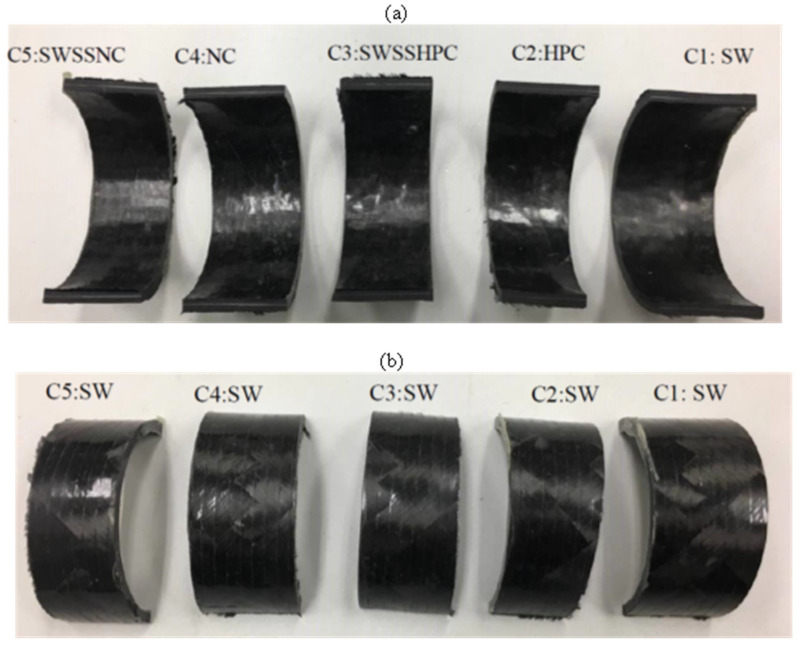
(**a**) Internal and (**b**) external surface change of CFRP tubes after 6-month exposure to the dual environments (Table 2).

After the 6-month exposure to C4 (NC internally and SW externally) and C5 (SWSSNC internally and SW externally), the inner surface of BFRP (Figure 4) and GFRP (Figure 5) exposed to the higher alkalinity solutions (i.e., NC, SWSSNC) had turned brighter and rougher, whereas only slight surface changes were observed on the outer surface of the tubes. Tubular specimens exposed to C1 (SW internally as well as externally), C2 (HPC internally and SW externally) and C3 (SWSSHPC internally and SW externally) did not exhibit any distinguishable surface changes. Regarding CFRP tubular specimens, the lustre and colour of both exposure surfaces largely remained unchanged, irrespective of the solution combinations they were exposed to (Figure 6).

Unlike the dual exposure (C1, C2 and C3), Guo et al. [3] found that the exposure of both sides of GFRP and BFRB to the simulated concrete solutions of NC, HPC, SWSSNC and SWSSHPC turned the surface of the immersed specimens brighter with a rough surface texture.

### 3.2. pH Changes

**Figure 7:**
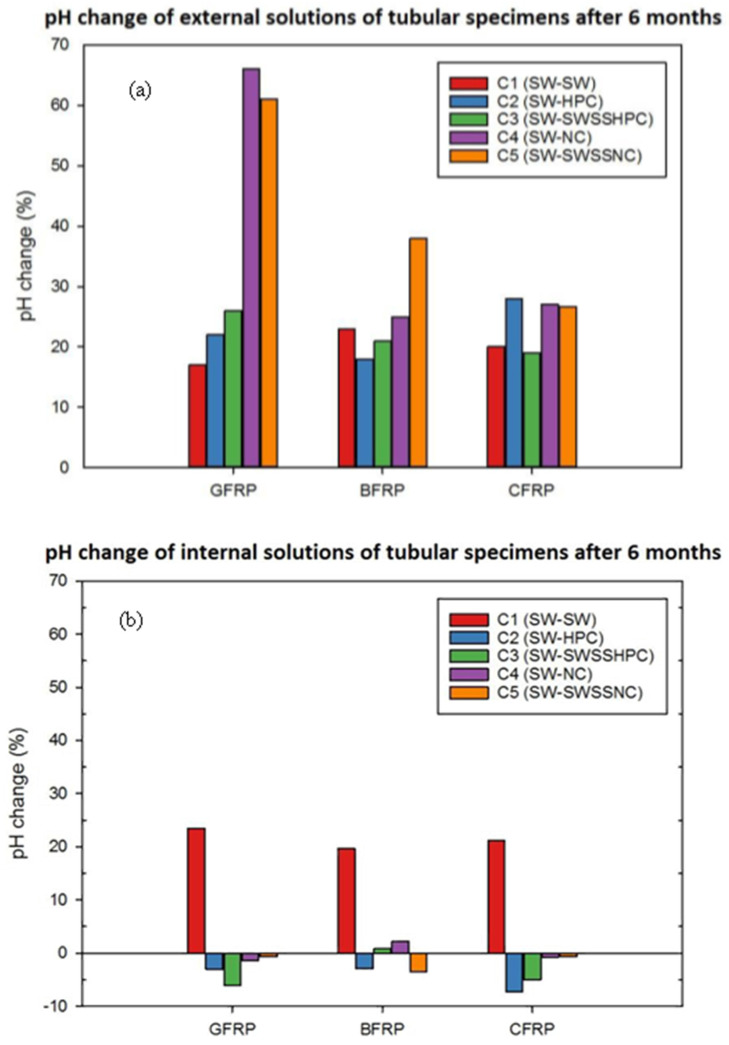
pH changes of (**a**) external and (**b**) internal solution after 6-month exposure of FRPs (e.g., C2 (SW-HPC) = SW externally and HPC internally).

The pH of both internal and external solutions of the FRP tubular specimens after 3- and 6-month exposure at 60 °C are summarised in Table 4, and a comparison of 6-month pH changes for CFRP, GFRP and BFRP is present in Figure 7.

After 3-month exposure, it is noticeable that the pH of the external solution in all cases (i.e., seawater, original pH~7.5) increased by at least 20%, and the greatest increase was found in the case where SWSSNC was the internal environment and seawater was the external environment (i.e., C5 condition in Table 2) for all types of FRPs. For the pH of internal solutions, they largely remained unchanged in all cases except for the reference exposure combination C1, which exhibited 20–30% pH increases of its internal seawater solution. Similar trends were found after 6-month exposure for both internal and external solutions in most cases. 

For pH increases of both the internal and external seawater solution in C1 for all FRPs, the most likely reason is the leaching of FRP component(s) during exposure in this specific environment. In other cases, particularly NC (C4) and SWSSNC (C5) internally for GFRP and BFRP, the considerably greater pH increase in the external solutions can be attributed to a greater leaching of alkalis from glass/basalt fibres and/or a diffusion of alkali ions from the internal alkaline solutions to the external solutions, which can be facilitated by an intensive chemical attack from the internal side of FRP tubes. Consistent with the visual observation (Section 3.1), the higher alkalinity solutions (i.e., NC, SWSSNC) tended to degrade glass/basalt fibres and the fibre–resin interface much more severely, which caused a greater transport of and uptake of moisture. This can also explain the relatively lower pH change of the external solution of CFRP, since carbon fibres and the fibre–matrix interface have been found to demonstrate good/durable degradation resistance in neutral pH solutions (SW) or higher alkalinity solutions (NC/SWSSNC) in the single environment exposure tests [3].

### 3.3. Attenuated Total Reflectance–Fourier Transform Infrared Spectroscopy (ATR-FTIR)

**Figure 8:**
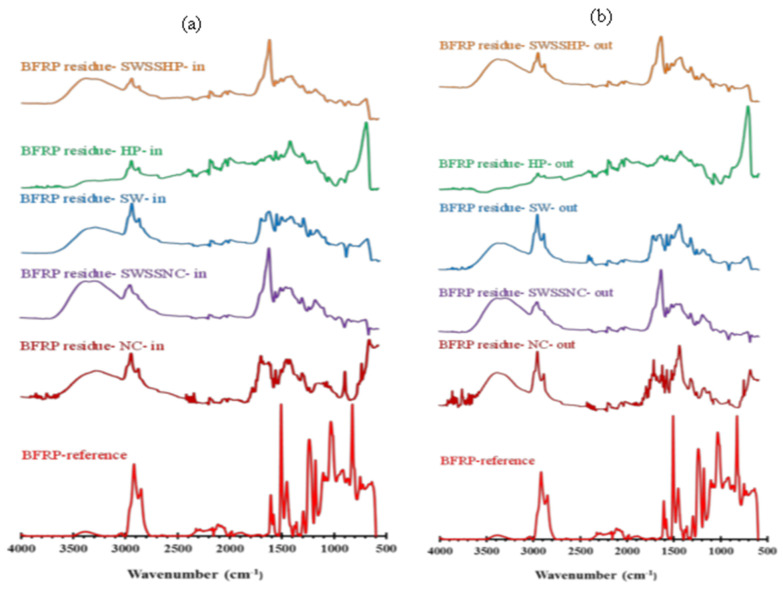
FTIR absorption spectra for the reference specimens and the (**a**) internal and (**b**) external solution residues after exposure of BFRP at 60 °C for 6 months.

**Figure 9:**
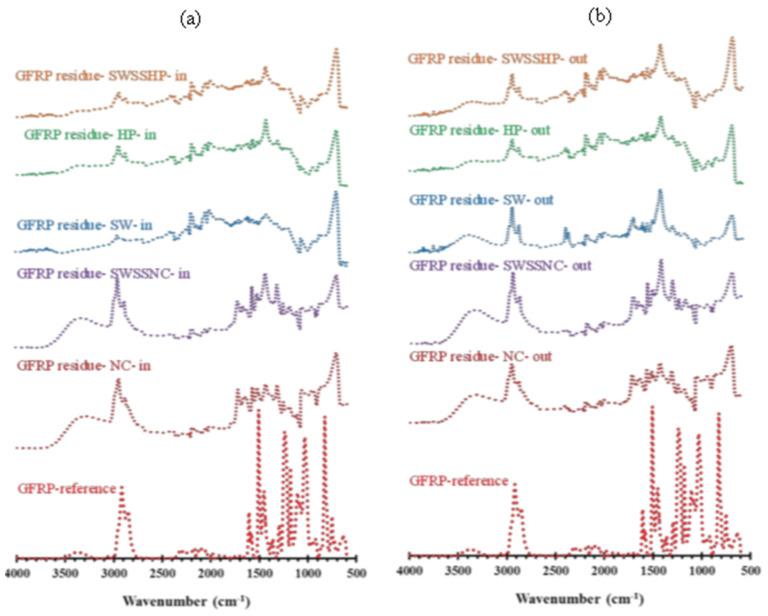
FTIR absorption spectra for the reference specimen and the (**a**) internal and (**b**) external solution residues after exposure of GFRP (E-glass) at 60 °C for 6 months.

**Figure 10:**
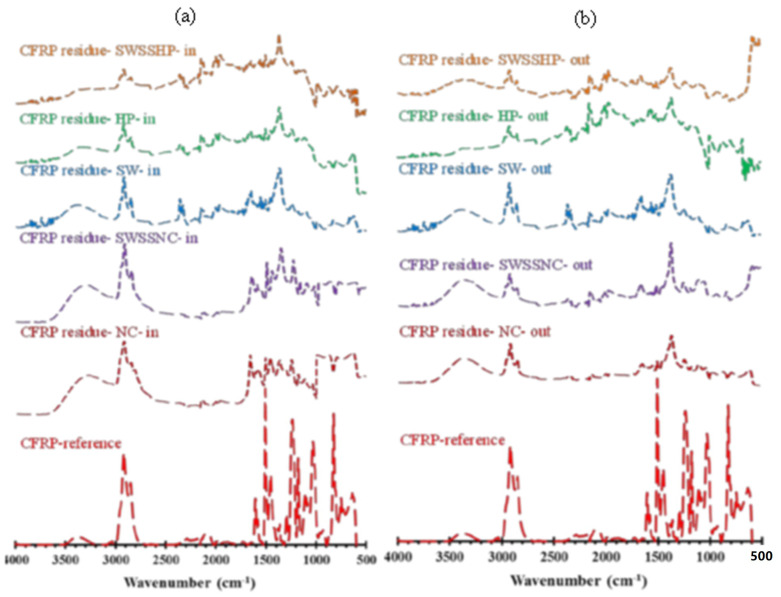
FTIR absorption spectrum for the reference specimens and the (**a**) internal and (**b**) external solution residues after exposure of CFRP at 60 °C for 6 months.

Figure 8, Figure 9 and Figure 10 present the absorption spectra of unexposed BFRP, GFRP and CFRP composites and the solution residues collected after the exposure of these three types of FRPs to five different dual-exposure combinations (Table 2) for 6 months at 60 °C. The assignments of the characteristic absorption bands of unexposed FRPs are provided in Table 5.

In the FTIR spectra of BFRP exposure solution residues (Figure 8), three new peaks at ~ 600 cm^−1^, ~840 cm^−1^ and ~1125 cm^−1^ were observed for all exposure combinations. The latter two peaks, which correspond to the changes in the vibration characteristics of the ether group and the vibration characteristics of the C-OH band, respectively [21], were found on the FTIR spectra for 6-month exposure solution residues in the single exposure environment test, whereas the peak at ~600 cm^−1^ corresponding to the Si-H and/or Fe-O bonds [22,23] was not observed in the single exposure environment test [3]. Only in the case of C4 (i.e., NC as the internal solution), a new stretching peak at ~699 cm^−1^ corresponding to the of Si-O bond was observed, which is probably due to the reaction of alkali ions with silicate on the fibres [3,24,25]. In addition, the O-H stretching of the alcohol peak at ~1415 cm^−1^, C=C stretching peak of alkene at ~1610 cm^−1^ and the C=C of the aromatic nucleus at 1509 cm^−1^ were observed to be more pronounced in all exposure combinations. The intensity increase of the O-H stretching peak (~3400 cm^−1^) may be attributed to the embedding of water (or hydroxyl group) with matrix resin during exposure. Other bands similar to those in the reference spectra for FRPs (e.g., stretching vibration of C-H group at 2930 and ~2900 cm^−1^) also appeared on the residue spectra, indicating the leaching of composite resin into both the internal and external exposure solution after 6 months.

In comparison, in the FTIR spectra of both the internal and external exposure solution residues of GFRP (Figure 9), the Si-H bending vibration peak at ~600 cm^−1^ and stretching of Si-O bond at ~700 cm^−1^ are more pronounced, which may indicate a greater degradation of its silicate content of the fibre. However, the intensities of other peaks (i.e., ~840, ~1125, ~1415, ~1610, 1509, 2930, ~2900 and 1300 cm^−1^) were observed to be less than those observed for BFRP exposure solution residues. 

In terms of the FTIR spectra for the exposure solution residues of CFRP tubular specimens (Figure 10), most peaks (e.g., ether group vibration ~840 cm^−1^, C-OH vibration at ~1125 cm^−1^) were the same as those on the spectrum of BFRP and GFRP (E-glass) exposure solution residues, except for the peaks of the Si-H and Si-O groups. In addition, a tiny peak at ~ 650 cm^−1^ appeared on the spectra, which is assigned for the O–H out-of-plane bending [26]. The intensities of peaks observed on the spectra of external solution residues appeared to be less pronounced than those on the spectra of internal solution residues, which might indicate that a greater resin leaching occurred internally.

### 3.4. Scanning Electron Microscopy (SEM)

**Figure 11:**
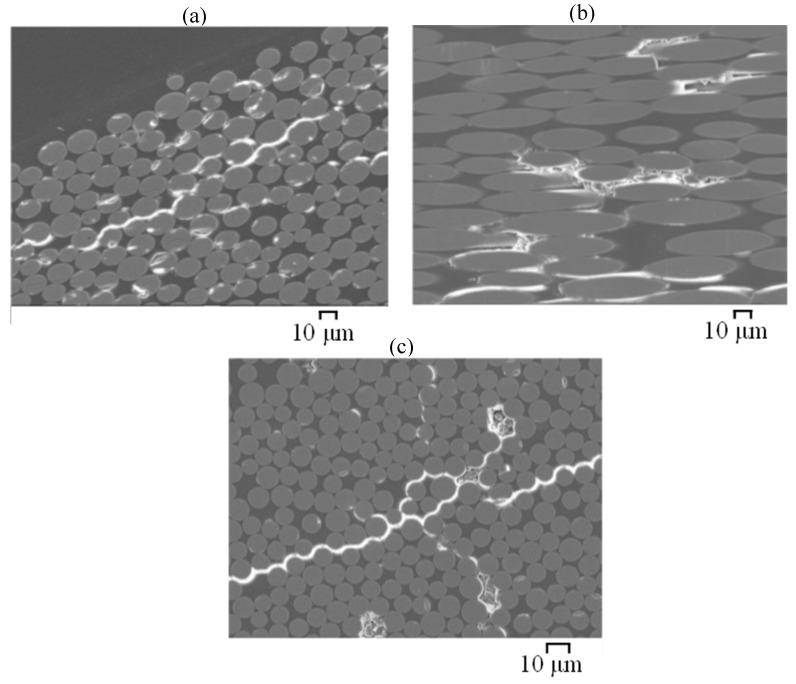
SEM images of the cross-sections of BFRP exposed for 6 months at 60 °C to C5 dual environment (Table 2): (**a**) adjacent to external surfaces; (**b**) adjacent to internal surface; (**c**) in the mid-width area (specimens prepared by grinding-and-polishing method).

**Figure 12:**
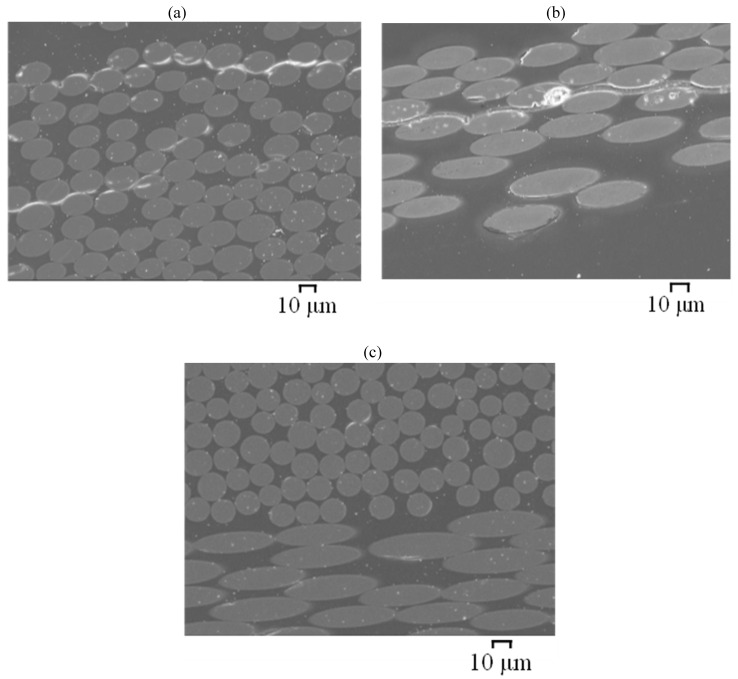
SEM images of the cross-sections of GFRP (E-glass) exposed for 6 months at 60 °C to C5 dual environment (Table 2): (**a**) adjacent to external surfaces; (**b**) adjacent to internal surface; (**c**) in the mid-width area (specimens prepared by grinding-and-polishing method).

**Figure 13:**
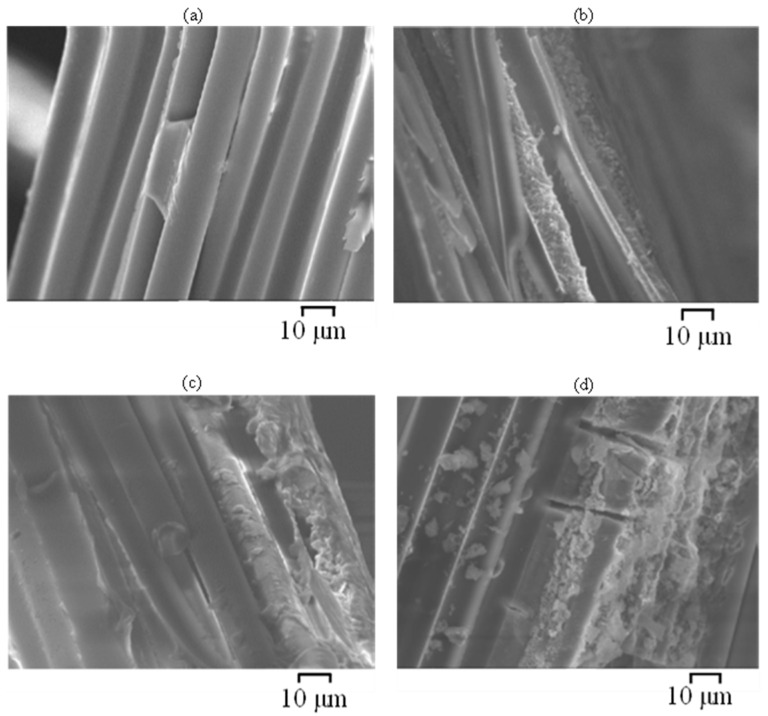
SEM images of the cross-sections of BFRP and GFRP (E-glass) exposed for 6 months at 60 °C to C5 dual environment (Table 2): (**a**) BFRP adjacent to external surfaces; (**b**) BFRP adjacent to internal surface; (**c**) GFRP adjacent to external surfaces; (**d**) GFRP adjacent to internal surface; (specimens prepared by liquid nitrogen fracturing method).

Cross-sections and fibre–matrix interfaces of GFRP and BFRP exposed to plain SW (C1) and SWSSNC (C5) were observed by SEM to examine their microstructural degradation after exposure. After exposure to SWSSC internally and SW externally (C5) for 6 months, the chemical degradation along the fibre–resin interface, combined with interface debonding (giving an appearance of continuous cracks), were observed adjacent to the external and internal exposure surfaces on a flattened cross section of the BFRP (Figure 11a,b) and GFRP (Figure 12a,b) specimens. They were observed even deeper into the centre of the BFRP specimen (Figure 11c), which indicates a deeper penetration of exposure solutions, more likely the internal SWSSNC solution, into the specimen. However, for the specimens fractured in liquid nitrogen, the fibres at the external edges (Figure 13a) and the centre of the BFRP were observed to remain clean and smooth, whereas only the fibres adjacent to the internal surface (Figure 13b) were observed to have degraded. For GFRP, the fibre degradation was found adjacent to both the external (Figure 13c) and internal surfaces (Figure 13d) but not deep in the interior. The observed fibre degradation near the external surface of GFRP that was exposed to SW could be attributed to a greater pH increase in the external solution (from ~7.5 to ~12; Table 3), and it agrees with the findings of the FTIR results of the GFRP (E-glass) described in the previous section.

Scrutiny of Figure 13 suggests that the corrosion shell and pitting are the main degradation features of the degraded glass and basalt fibres in the SWSSNC environment. Guo et al. [3] reported that such features could be observed on glass/basalt fibres just after a few days, or even in a few hours of exposure to the alkaline solutions. Such observations are attributed to the reaction of silicate in fibres with alkali-ions in the concrete solution (Equation (1)) [27,28,29,30,31]:

(1) $$\equiv \mathrm{Si}-\mathrm{OR}+\left(H^{+}+{\;\mathrm{OH}}^{-}\right)\;\to \;\equiv \mathrm{Si}-\mathrm{OH}+R-\mathrm{OH}$$

The chemical reaction that describes the successive degradation and gradual disruption of the silicate network is shown in Equation (2) [27,28,29,30,31]:

(2) $$\equiv \mathrm{Si}-O-\mathrm{Si}+\left(R^{+}+{\;\mathrm{OH}}^{-}\right)\;\to \;\equiv \mathrm{Si}-\mathrm{OH}+\mathrm{RO}-\mathrm{Si}$$

Further observations by SEM (Figure 13) suggest that the greater extent of fibre and fibre–matrix interface degradation is to occur adjacent to the internal surface of the BFRP and GFRB during the exposure to SWSSNC (as compared to the external surface exposed to SW). Besides the degradation of silicate in the fibres (Equations (1) and (2)), aluminium oxide reacts with alkali ions in the alkaline environment (pH > 9) according to the reaction described in Equation (3) [32,33]:

(3) $${\mathrm{Al}}_{2}O_{2\;}+2{\mathrm{OH}}^{-}{}_{\left(\mathrm{adsorbed}\right)}\;\to 2{\mathrm{AlO}}_{2}{{}^{-}}_{\left(\mathrm{aqueous}\right)}+H_{2}O$$

Aluminium ions in the fibres react with chloride ions from the SWSSC, forming soluble oxychloride complexes $(\mathrm{Al}\left(\mathrm{OH}{)}_{2}{\mathrm{Cl}}_{2}{}^{-}\right)$ as shown by Equation (4) [34]:

(4) $${\mathrm{Al}}^{3+}{}_{\left(\mathrm{crystal}\;\mathrm{lattice}\;\mathrm{of}\;\mathrm{the}\;\mathrm{oxide}\right)}+2{\mathrm{Cl}}^{-}+2{\mathrm{OH}\;}^{-}\to \mathrm{Al}{\left(\mathrm{OH}\right)}_{2}{\mathrm{Cl}}_{2}$$

Consequently, the soluble oxychloride complexes are leached out to the simulated concrete solutions. In the chloride solution, Mg oxide/hydroxide also dissolves and leaches into the corrosive solution [32].

For the basalt fibre, the ferric hydroxide forms due to the hydrolysis of ferric iron according to Equation (5) [35], where the rate of hydrolysis increases in the alkaline solution [36]:

(5) $${\mathrm{Fe}}^{3+}+3{\mathrm{OH}}^{-}\;\to \mathrm{Fe}{\left(\mathrm{OH}\right)}_{3\;\left(\mathrm{aqueous}\right)}$$

As the Cl^−^ ions are present in SWSSNC, the ferric irons react with chloride ions forming the complex of iron chloride [37]:

(6) $${\mathrm{Fe}}^{3+}{}_{\left(\mathrm{aqueous}\right)}+{\;\mathrm{Cl}}^{-}{}_{\left(\mathrm{aqueous}\right)}↔\mathrm{Fe}\left(H_{2}O\right){\mathrm{Cl}}^{2+}{}_{\left(\mathrm{aqueous}\right)}$$

(7) $$\mathrm{Fe}\left(H_{2}O\right){\mathrm{Cl}}^{2+}{}_{\left(\mathrm{aqueous}\right)}\;↔{\;\mathrm{FeCl}}^{2+}{}_{\left(\mathrm{aqueous}\right)}$$

(8) $${\mathrm{FeCl}}^{2+}{}_{\left(\mathrm{aqueous}\right)}+{\;\mathrm{Cl}}^{-}{}_{\left(\mathrm{aqueous}\right)\;}\to {\;\mathrm{FeCl}}^{2+}{}_{\left(\mathrm{aqueous}\right)}$$

(9) $${\mathrm{FeCl}}^{2+}{}_{\left(\mathrm{aqueous}\right)}+{\;\mathrm{Cl}}^{-}{}_{\left(\mathrm{aqueous}\right)\;}\to {\;\mathrm{FeCl}}_{3\;\left(\mathrm{aqueous}\right)}$$

The higher degradation rate of the fibres and fibre/matrix of the internal surfaces of the GFRP and BFRP during exposure to the SWSSNC could play a major role in leaching out of the alkaline solution toward the outer surface exposed to the SW environment, as suggested by the significant increase in pH in the SW (Figure 7a and Table 2).

**Figure 14:**
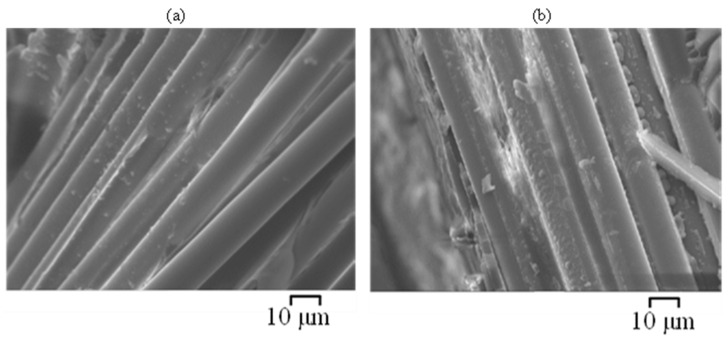
SEM images of the cross-sections of (**a**) BFRP and (**b**) GFRP (E-glass) exposed for 6 months at 60 °C to C1 environment (Table 2) (specimens prepared by liquid nitrogen fracturing method).

For specimens exposed to C1 (i.e., exposure to SW both internally and externally), minor interface debondings can be observed adjacent to the surface of the GFRP/BFRP specimens, prepared by the grinding-and-polishing method. On a fracture surface, the majority of the basalt fibres were observed to have remained clean and smooth except for a few fibres adjacent to the exposure edges where signs of pitting and some particle deposition were found (Figure 14a). However, glass fibres adjacent to surfaces were found to have slightly degraded (Figure 14b), as some corrosion shell-like patches adherent on the fibre surface and plate-shape-like corrosion products were observed.

## 4. Conclusions

Fibre-reinforced polymers (FRPs) have a great potential for their use as confining tubes for concrete-filled tubular structures in a marine environment where such structures use seawater sea sand concrete (SWSSC). Such an application will expose FRP tubes to a dual environment of seawater (SW) externally and SWSSC internally. This study explored the effects of such dual environment exposure on the durability performance of E-glass-fibre-reinforced polymer (GFRP), basalt-fibre-reinforced polymer (BFRP) and carbon-fibre-reinforced polymer (CFRP). The key findings are summarised below:

1. Exposure of epoxy-based FRPs to seawater resulted in an ~20% pH increase in the exposure solution, which is attributed to the leaching of the polymer resins. A dual exposure environment of the simulated concrete pore solution internally, and the seawater externally, resulted in a greater pH increase in the external solution.
2. For GFRP/BFRP exposed to seawater, both internally and externally, minor interface debondings were observed near the exposure surfaces, and basalt fibres largely remained clean and smooth, whereas glass fibres near the exposure surfaces were found to have slightly degraded.
3. For BFRP and GFRP (E-glass) that were exposed to a dual environment of SWSSNC internally and SW externally, fibre–resin interface debondings joined to give the appearance of continuous cracks adjacent to both the internal and external surfaces as well as in the centre of the BFRP. Although a clear progressive degradation pattern cannot be identified from the ground-and-polished cross sections of the specimens, possibly due to the surface damages caused by the mechanical process, the fibre degradation was generally observed on fractured specimens. Basalt fibres were observed to have degraded adjacent to the internal exposure surface, whereas fibres at the rest parts remained clean and smooth; glass fibre degradation was found adjacent to both surfaces of the GFRP. The greater fibre degradation adjacent to the external surface of the GFRP (E-glass) than the BFRP might be attributed to a greater pH increase in the external exposure solution of the GFRP (E-glass) in this exposure environment of SWSSNC internally and SW externally.
4. CFRP exhibited the best durability in a dual exposure environment of seawater sea sand normal concrete (SWSSNC) internally and seawater externally.

## Figures and Tables

**Table 1:** Chemical compositions of E-glass and basalt fibers [3].

Types	Chemical Composition (wt%)												
	SiO_2_	Al_2_O_3_	Fe_2_O_3_	CaO	MgO	Na_2_O	K_2_O	TiO_2_	MnO	P_2_O_5_	ZnO	CuO	BaO
E-glass	46.7	10	0.31	17.1	2.09	0.30	0.27	0.23	0.01	0.04	0.01	<0.01	0.02
Basalt	46.7	14.6	8.51	6.69	2.83	2.07	1.31	0.97	0.14	0.16	0.04	0.01	0.03

**Table 2:** Combinations of dual environments of tubular specimens.

Exposure Environment		
Specimen Identification	Internal Solution	External Solution
C1	SW	SW
C2	HPC	SW
C3	SWSSHPC	SW
C4	NC	SW
C5	SWSSNC	SW

**Table 3:** Chemical composition and pH of test solutions.

Test Solutions	Simulated Environment	Test Solution Composition (g/L)				pH
		NaOH	KOH	Ca(OH)_2_	NaCl	
1 a	SWSSNC	2.4	19.6	2.0	35	~13.4
2 b	NC	2.4	19.6	2.0		~13.4
3 a	SWSSHPC	0.6	1.4	0.037	35	~12.7
4 b	HPC	0.6	1.4	0.037		~12.7
5	DW					~7.5

a: Simulated SWSSNC and SWSSHPC pore solutions were prepared according to [3,16,17,18]. b: NC and HPC simulated pore solutions were prepared according to [19,20].

**Table 4:** pH change of internal (I) and external (E) solutions of FRP tubes after 3- and 6-month exposures.

Solutions Original pH				3 Months pH				6 Months pH			
		**E**	**I**	**E**	**E-C**		**I-C**	**E**	**E-C**	**I**	**I-C**
**GFRP**	Seawater (C1)		7.5	9.33	+24%	9.65	+29%	8.80	+17%	9.26	+23%
	HPC (C2)		12.7	9.46	+26%	11.98	−6%	9.16	+22%	12.31	−3%
	SWSSHPC (C3)	7.5	12.7	10.25	+37%	12.36	−3%	9.46	+26%	11.92	−6%
	NC (C4)		13.4	11.04	+47%	13.37	0%	12.43	+66%	13.22	−1%
	SWSSNC (C5)		13.4	11.81	+57%	13.20	−1%	12.08	+61%	13.31	−1%
**BFRP**	Seawater (C1)		7.5	9.17	+22%	9.40	+25%	9.26	+23%	8.98	+20%
	HPC (C2)		12.7	9.27	+24%	12.64	−1%	8.83	+18%	12.32	−3%
	SWSSHPC (C3)	7.5	12.7	9.23	+23%	12.27	−3%	9.11	+21%	12.8	1%
	NC (C4)		13.4	9.44	+26%	13.20	−1%	9.35	+25%	13.70	2%
	SWSSNC (C5)		13.4	10.60	+41%	13.13	−2%	10.32	+38%	12.94	−3%
**CFRP**	Seawater (C1)		7.5	9.11	+21%	9.39	+25%	9.03	+20%	9.09	+21%
	HPC (C2)		12.7	9.40	+25%	12.46	−2%	9.61	+28%	11.78	−7%
	SWSSHPC (C3)	7.5	12.7	9.47	+26%	12.30	−3%	8.89	+19%	12.07	−5%
	NC (C4)		13.4	9.46	+26%	13.58	+1%	9.55	+27%	13.30	−1%
	SWSSNC (C5)		13.4	9.51	+27%	13.48	+1%	9.50	+27%	13.31	−1%

Note: E = External solution; I = Internal solution; E-C = External solution pH change; I-C = Internal solution pH change; ‘+’ = increase; ‘−’ = decrease.

**Table 5:** Characteristic absorption bands observed on unexposed FRP specimens.

Adsorption Bands (cm^−1^)	Assignment
3400	O-H stretching band
~2930 and ~2900	Stretching vibration of C-H group
~1610	C=C stretching band (alkane)
~1509	C=C (aromatic nucleus)
~1415	O-H stretching (alcohol)
~1300	C-N stretching band
~1245	Asymmetric stretching vibration C-O-Φ
~1220	C-O-H (of guaiacyl ring)
~1040	symmetric stretching vibration C-O-Φ
~827	Out of plane bending of C-H (benzene)
~800	CH_2_ rocking

## Data Availability

Data is contained within the article.
